# Design and Customization of Telemedicine Systems

**DOI:** 10.1155/2013/618025

**Published:** 2013-05-15

**Authors:** Claudia I. Martínez-Alcalá, Mirna Muñoz, Josep Monguet-Fierro

**Affiliations:** ^1^Multimedia Engineering Department, Universitat Politècnica de Catalunya, ETSEIB, Avenida Diagonal 647, Barcelona, Spain; ^2^Centro de Investigación en Matemáticas, Avenida Universidad No. 222, 98068 Zacatecas, ZAC, Mexico

## Abstract

In recent years, the advances in information and communication technology (ICT) have resulted in the development of systems and applications aimed at supporting rehabilitation therapy that contributes to enrich patients' life quality. This work is focused on the improvement of the telemedicine systems with the purpose of customizing therapies according to the profile and disability of patients. For doing this, as salient contribution, this work proposes the adoption of user-centered design (UCD) methodology for the design and development of telemedicine systems in order to support the rehabilitation of patients with neurological disorders. Finally, some applications of the UCD methodology in the telemedicine field are presented as a proof of concept.

## 1. Introduction

In recent years, the advances in information and communication technology (ICT) have enabled the development of systems and applications aimed at supporting rehabilitation therapy and, therefore, contributing to the enrichment of patients' life quality [1–3]. The creation and implementation of web-enabled communication, patient services, and other e-Health initiatives have been significantly developed and enhanced in order to improve the quality of health services and maintain a competitive advantage. Consequently, the quality of health care has significantly improved [4, 5]. Traditionally, technology has supported health professionals by providing instruments, diagnosis, and different therapeutic treatments [6]. Moreover, the information and communication technologies have expanded their application to management and planning activities of health areas. Thus, companies involved in the health area must expand their capabilities to all stakeholders, including patients and the public in general towards robust, efficient, and friendly telemedicine systems [7, 8].

According to the literature [9, 10], doctors and nurses make use of the Internet in two mainly ways: (1) for communication, to send information through email, and (2) as an extensive library, to consult the clinical information. Also, it is mentioned that they have good computer skills, a positive attitude towards using the computer and Internet, and are motivated to use both ways on daily activities. However, [11] mentioned that some health professionals still show some resistance towards the acceptance of new technologies, even when some health sectors are beginning to integrate ICT in some of their fields. 

Besides, Bernard et al. [12] mention that ICTs offer practical and timely mechanisms for continuing medical education allowing the improvement of educational programs for health professionals in rural areas [13–15]. Simultaneously, ICT may also have an important role in transferring clinical data [16]. The American Psychiatric Association (APA) states that once the information is stored, it is essential to have access to it. Moreover, recent technological advances have enabled the introduction of a broad range of telemedicine applications, such as teleradiology, teleconsultation, telesurgery, remote patient monitoring, and health-care records management that are supported by computer networks and wireless communication [17–20].

Through the development of user interfaces for health-care applications, researchers have empirically evaluated the effectiveness of diverse user-centered design (UCD) approaches [21, 22]. Health-care software developers often overlook relevant user features, user tasks, user preferences, and usability issues. Thus, systems can provoke a decrease in productivity or simply be unusable. Several factors could be attributed to developing poor systems, such as cost and time restrictions and the lack of developers with sufficient knowledge on user-centered design [23].

According to the literature [24, 25], only 61% of information system projects meet the customer requirement specifications. Furthermore, 63% of projects exceed their estimated budgets due to the inadequate initial user analysis [24]. Incorporating good design principles in the beginning phase of a project not only saves time and cost, but also decreases changes in design late in the development process [25].

**Figure 1:**
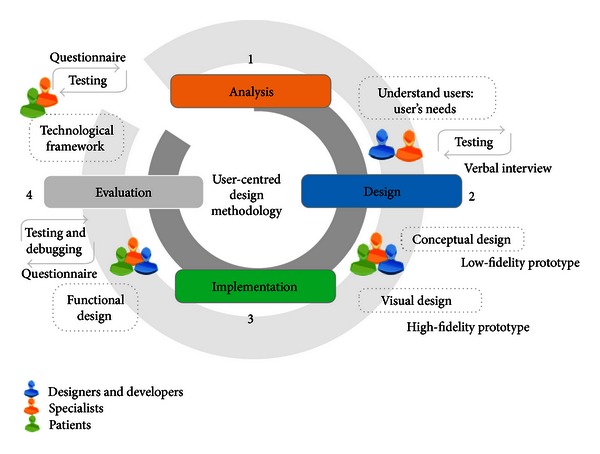
Design process of the telemedicine systems centered on the user. Adaptation of Martínez Alcalá [50].

According to Wallach and Scholz [26], a UCD is defined as a design process and evaluation that pays attention to the intended user, focusing on what they will do with the product, where they will use it, and what features they consider essential. In particular, a UCD may be used by designers to address the needs of the patients and specialists about questions related to experiences with mental models of illness and disabilities.  Figure 1 provides a graphical illustration of the process of UCD used in this research work.

As Figure 1 shows, this research presents a user centered design framework (UCD), specifically for customizing the design of health interfaces. The UCD contains four steps to follow: (1) analysis, (2) design, (3) implementation, and (4) evaluation. The goal of customizing the design of the interfaces is to model and develop systems based on the user needs and features. Therefore, the UCD used proposes to involve patients and specialists throughout the design phases, leading to easy-to-learn systems that increase user productivity and satisfaction and user acceptance and that reduce user errors and user training time.

## 2. State of the Art

Broadly, telemedicine refers to the use of information and telecommunication technologies to distribute information and/or expertise necessary for healthcare service provision, collaboration, and/or delivery among geographically separated participants, including physicians and patients [27, 28]. Different definitions highlight that telemedicine is an open and constantly evolving science, as it incorporates new technological advancements, and it responds and adapts to the changing societies' health needs [29]. Telemedicine supports different types of relationship between two or more actors who are not in a common physical space. The most common relationships established are (1) professional-professional, (2) professional-professional-patient, and (3) professional-patient.

Telemedicine covers different forms of information: (1) transmission (voice, sound, video, still picture, and text); (2) communication technologies (standard telephone lines, coaxial cable, satellite, microwave, digital wireless, ISDN, and Internet); and (3) user interfaces (desktop computers, laptop computers, personal digital assistants, fax machines, telephones, mobile phones, videophones, various stand-alone systems, and peripheries). All of them allow to carry out a wide range of activities, such as store-and-forward applications, which involve the asynchronous transmission of medical information, patient/health provider communications, and other data, and live audiographic encounters, which combine sound with still pictures, and perhaps, most importantly, the live two-way interactive video consultation [30, 31].

One of the main motivations for the application of ICT in both healthcare organizations, public and private, lies in the necessity of improving the information and providing medical care to a multitude of geographically dispersed agents. Clinical studies have shown that telemedicine is safe and cost-effective, compared with hospital treatment, especially with patients suffering from chronic diseases [32]. Besides, it is important to highlight that the introduction of telemedicine services must overcome a series of obstacles such as acceptance by patients, accessibility issues, technology costs, physical and psychological disabilities of patients, and acceptance and availability of medical personal [33, 34]. Despite all obstacles and failures, telemedicine concepts have been considered of great potential to support healthcare in particular for patients with neurological diseases. Since most of these patients are from older age groups, it is important to develop concepts, systems, and devices that can be handled by older patients and customized to their individual needs and limitations.

In this context, it is important to mention that despite its growth, there is a general feeling that telemedicine has a long way to go before it reaches its maximum potential [35]. Therefore, many different telemedical systems have been developed and used on various scales simply as experimental until a broader routine is carried out. Each of these scale systems has been more or less successful. However, the development of telemedicine systems and devices has often been defined by technical possibilities rather than by the needs of patients or their caregivers. Then, in order to know the performance of a telemedicine system, it needs to be evaluated not only for their benefit, but also for their feasibility, acceptance, and economic efficiency. Moreover, studies focused on telemedicine should consider research scenarios close to rehabilitation and should include older patients and patients with cognitive and physical limitations [35].

### 2.1. Advantages and Disadvantages with respect to Other Systems Developed

Table 1 summarizes the major advantages and disadvantages of other existing telemedicine systems.

## 3. Materials and Methods

### 3.1. Reference Model for the User-Centered Design of the Telemedicine Systems

The design process employed in the development of the telemedicine systems was inspired on the user-centered design (UCD) approach, a widely accepted methodology for creating usable applications or systems, which aims to truly meet the needs of users [3].  Figure 1 gives an overview of the developed method. The method is composed of four phases: (1) analysis, (2) design, (3) implementation, and (4) evaluation. The method phases are further described below.

The main challenge of this method is the customization of activities according to the user's needs. Besides, this approach can be achieved only when the user is actively involved during the design and evaluation of an application or system.

The following sections describe each of the method phases.

*(1) Analysis.* This phase is focused on three variables: (1) user features, which include cognitive functioning and disabilities, (2) activities characteristics, which include the definition and gathering of content, and (3) definition of the profiles, which includes the identification of end user. (The end user is a user that interacts with a specific device, system, or service. The main users of healthcare ICT are (a) healthcare professionals who work with healthcare information and communication technology applications in hospitals and other healthcare organizations, (b) patients, (c) the general public (regarding the eHealth services), and (d) other supportive parties (e.g., parents, family, and social care workers).)

Then, this phase is focused on each user. The phase begins interviewing patients and specialists to know the information that should be reflected in the system. Then, the objectives that each activity must achieve are identified. Afterwards, the user profiles, which allow the customization of activities and therapy according to the user, are defined. Finally, the analysis and definition of the therapy model are developed.

*(2) Design.* This phase is focused on the conceptualization of user requirements. This phase begins designing low-fidelity prototypes, which show essential elements of the interface for a specific user. Then, the high-fidelity prototypes, which consist of developing visual design of each end user interface, are developed. Each prototype is assessed by all users to identify any discrepancies or errors during the implementation phase.

*(3) Implementation.* This phase is focused on creating a layout that includes all the navigation features for each system component (activities, therapies, and progress). The design and implementation phases are iterative. Before implementing a real environment with real users, the prototypes, two things happen: the functional designs are evaluated by all users, and errors that have been identified are corrected and debugged.

*(4) Evaluation.* This phase is focused on developing the application according to the proposed design and its improvements that resulted from consistent user feedback. The user feedback can be obtained during different stages of the design (conceptual design, visual design, and functional design). In this phase a prototype evaluation with end users is performed.

### 3.2. MAIA Framework

The MAIA framework is used to support the idea of institutions as a major structure for conceptualizing social systems. The MAIA framework extends and formalizes the components of the IAD4 (an institutional framework that provides a collection of concepts present in a social system with an institutional perspective) to present a metamodel for conceptualizing social systems for agent-based simulation [36].

The MAIA framework has been used in this research by designers with different professional profiles in order to conceptualize the components of telemedicine systems. According to Ferruzca Navarro [37], MAIA is a useful tool to identify those involved in a system. It represents the system through a graphical structure and builds a table to describe its proposed components.

Now, a conceptual model based on the framework for the analysis of interactions between agents (MAIA) is presented [37]. According to Ferruzca Navarro [37], the conceptual model behind this framework is composed of structural components and articulations. The interactions within a system lead to patterns that can be evaluated by the analyst. The MAIA framework views actors as institutional-driven entities. Agents form the key concepts of the modeled system and then are placed within a context.

The main objective of MAIA framework is the identification of the key players involved in a telemedicine system and their relationships.

**Figure 2:**
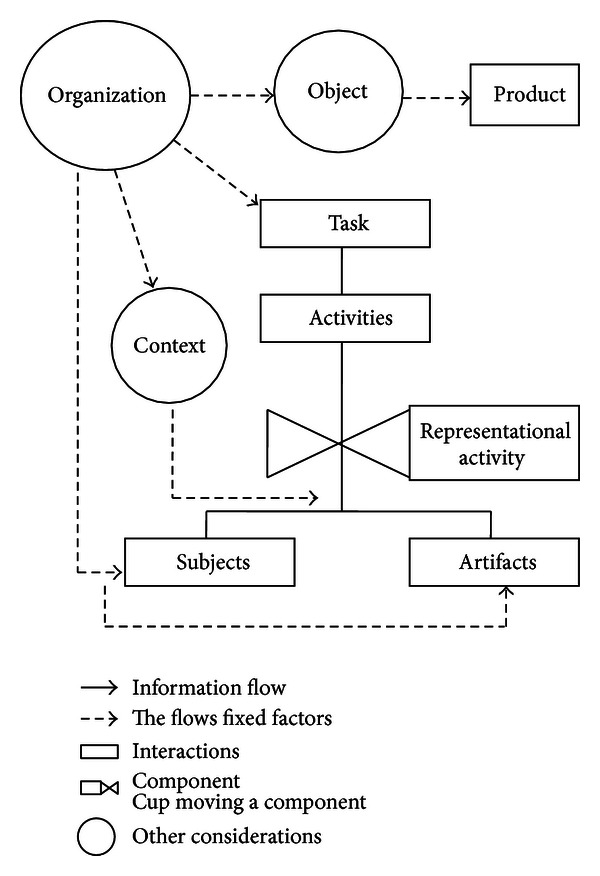
Conceptual model of the telemedicine system components [37].

The main components of MAIA are *organization, subject, artifacts, and context*, which are considered structural agents within the system that coordinate their actions according to the pursued objective, giving place to other elements such as *object, task activities, products, and representational activity* (see Figure 2).

A fundamental aspect of any system is the analysis and understanding of their composition. In this regard, the components (entities) related to the structure of the telemedicine system are described based on the MAIA model. According to the author in [37], the conceptual model of this methodology consists of structural components and articulation. Each is briefly described here.

#### 3.2.1. Structural Components

*(i) Organization. *The organization establishes the division of work in the system and their operating rules (procedures). Furthermore, it provides the means of communication and working that subjects have to use in their work. Examples of organization are hospitals, schools, research centers, and laboratories.

*(ii) Subject.* A subject is an agent who starts a task and is able to interact with other members of the system. The description of a subject can be based on demographics, physical or motor skills and cognitive, emotional, or affective aspects. A subject may perform one or more roles within the system. Examples of subjects include patients, families, caregivers, and specialists.

*(iii) Artifact. *It is a tangible or intangible resource that allows the execution of a task. The artifacts affect what subjects do and how they do it. There are many ways to understand the artifacts. Besides, there are several ways to describe the artifacts, such as their appearance, use, and personal satisfaction. Examples of artifacts include computers, mobile documents, equipment, and applications.

*(iv) Context: Workspace (Physical or Virtual) in Which the subjects and Artifacts Are Developed.* The context affects the learning process of individuals as well as their behavior. Examples of spaces include hospital therapy rooms, home, and web applications.

*(v) Product: Result of the Interactive Activity between the System Components in Accordance with the Objective Pursued. *A device, a service, and a professional are examples of products obtained through various tasks. A “product” could be employed in another system as artifact, procedure, subject, or environment.

#### 3.2.2. Articulation Components

*(i) Objective.* The organization raises a number of goals that must be achieved. These goals determine the behavior of the subjects. To achieve these goals, people perform tasks which lean on other people, artifacts, and environments.

*(ii) Task.* The fulfillment of objectives is performed by carrying out a series of tasks that are designed to achieve them. The tasks are assigned based on the role of each person or group and whose complexity can break down into a set of simple activities.

**Figure 3:**
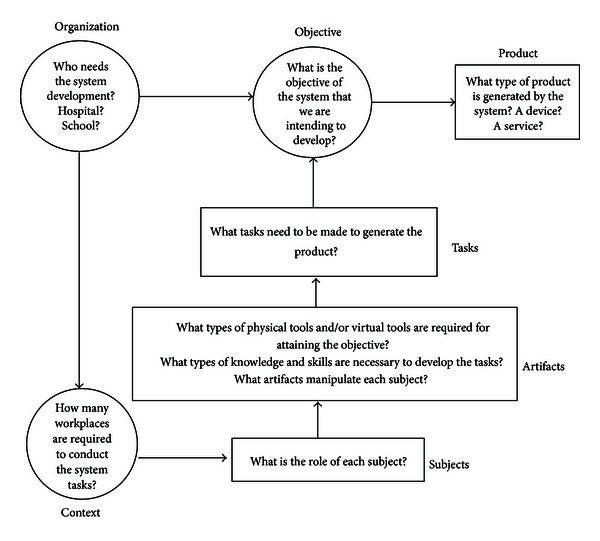
Questions guide to the identification of system components [37].

*(iii) Activity: Set of Steps to Carry Out a Task.* After defining the components of the MAIA framework, the structural components may be immediately identified to make up the system. Similarly, it is possible to define the articulation components. Next, we are showing the questions that guide the identification of system components (see Figure 3).

## 4. Results 

At the Laboratory of Multimedia Applications at the Universitat Politécnica de Catalunya, telemedicine systems have been developed. The telemedicine systems aim to create multimedia tools, available through the Internet, which contribute to improve patients and families life quality. The systems are intended not only to serve in the rehabilitation of patients, but also to therapists and specialists in the process of monitoring the therapy. Next a detailed description of the telemedicine systems created based on the reference model for UCD, described in the previous section, is presented.

### 4.1. Case Study

#### 4.1.1. Case Study 1: The eMental System

The main objective of the eMental system is to support the rehabilitation of the elderly with cognitive impairment and to promote their social integration. The eMental system provides a cognitive stimulation therapy to the patients, caregivers, and specialists. Its primary function is to improve the capabilities of people with any cognitive alteration, through a telemedicine system.

*(1) Analysis.* The degenerative disorders, which include cortical dementias such as Alzheimer's disease (AD) and subcortical dementias such as Parkinson's disease, are most prevalent. Cognitive impairments also result from other less prevalent conditions as traumatic brain injuries (TBIs); vascular disorders such as strokes; other progressive disorders of the central nervous system such as multiple sclerosis (MS); toxic conditions such as alcoholism; infectious processes such as HIV and AIDS; brain tumors; oxygen deprivation; and metabolic conditions such as diabetes. Cognitive decline also occurs routinely in individuals who are aging “normally” [38]. The patients with cognitive impairment have some physical and mental limitations, so they are sometimes assisted by their caregiver or family to perform daily activities.

As a result of a first approach with a hospital in Spain, it was possible to define the participation of different actors that were involved in the rehabilitation process. 

The eMental system is directed to the next users: doctors, patients, caregivers, and other specialists (see Table 2). Next, the approaches obtained from the study are presented as follows:

- professional cases (doctors, therapists, and other specialists) require a telemedicine system that helps them monitor and trace patients with cognitive alterations. It also should be able to manage customized therapy sessions;
- patient cases require an asynchronously system that allows patients to access from any point and place without going to the hospital or rehabilitation place. It allows health-care providers to follow the patient's evolution and medication at home and, in general, monitoring the patient from a distance;
- patients suffering from cognitive alterations such as brain injury, mild or moderate Alzheimer, neurodegenerative disorders, psychiatric disorder associated with cognitive impairment. This procedure includes supporting family and/or caregivers that assist in the rehabilitation of patients.

To understand the needs and limitations of each user, a direct observation on the subjects of the research (patients, caregivers, and specialists) was used focusing on their situation, environment, and activity. To do so, verbal interviews were performed to all users interested in research; this allows to collecting their opinions and experiences. Then, these comments were taken into account in the system design process.

**Figure 4:**
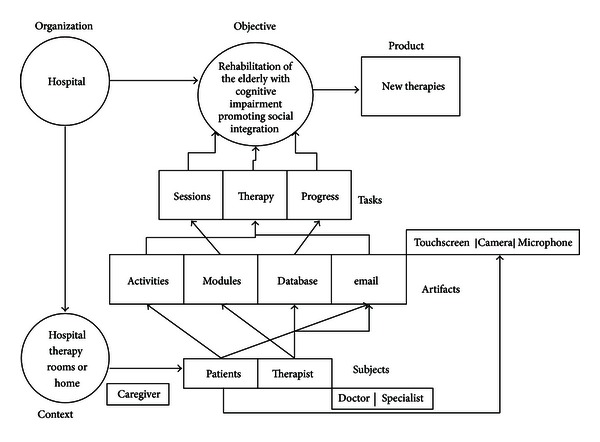
Representation of the therapy process for the eMental system by following MAIA methodology.

After understanding the users' goals and needs, the next step consisted in organizing the information according to the MAIA framework [37]. In order to facilitate the understanding of the main actors involved in the therapeutic process and to allow the development of further systems adapted to the actual needs and circumstances, the representation of the therapy process for the eMental system by following MAIA methodology is shown (see Figure 4).

*(2) Design.* The eMental system is designed for specialists in cognitive therapy. At the design and development process, different roles were identified as follows: (a) the medical team defined the contents; (b) the design team proposed the graphical user interface (GUI) (*Graphic User Interface* represents the information and actions available to a user through icons and graphical elements [39]) that attempted to represent communication between the patient and the specialist; and finally (c) the development team was responsible for programming and executing the contents of the system. The eMental system considered important capabilities such as attention and concentration, executive functions (reasoning, planning), perception and knowledge, language, and computation special orientation.

In order to characterize the contents of the system as far as possible, a conceptual design of the eMental system was schematized after conducting a literature review. This process was conducted with the support of several medical specialists and taking into account the opinions and experiences gathered in the direct observation. In the traditional therapy, the therapist performs the therapy through using pencil and paper supported with a picture book with cognitive stimulation exercises.

The eMental system was designed in a way that the therapy can be performed by the patient in the comfort of his or her home. Each of the cognitive stimulation exercises mentioned in the picture book were adapted and customized in the system so that patients could perform their exercises in a more comfortably and easily way. Furthermore, the therapists could perform the rehabilitation therapy more quickly and with a greater control over the results. This allows to attain a reduction of costs and time for both the patient and the hospital. To achieve this, the therapist classified each of the years in a total of 6 items, while the design team digitized each of the necessary fonts (*images, photographs, diagrams, etc.*) to design the exercises within the system.

Once the components and contents were defined, the team performed several low-fidelity prototypes (sketches) through designing quick and easy user interface. These prototypes were presented to the medical team and the development team in order to get comments and new ideas regarding the design of the system framework.

Therefore, a better designed high-fidelity prototype was obtained. Next, three important areas of the system are determined in order to understand the tasks, the therapy, and the progress. These are described as follows:

- the task area contains customized activities that the doctor or therapist considers relevant to the patient's rehabilitation;
- the therapy area has *n* modules that integrate memory exercises, numbers, letters, and drawings. It also has three levels which are adjusted to the patient's evaluation;
- the progress area: the results obtained during the therapy process can be displayed graphically to the users.

This eMental system consists of a series of 96 exercises such as calculation, memory, attention, orientation, language, and visuospatial exercises designed to address mental capacities like attention and concentration, executive functions (reasoning, planning), perception and knowledge, language, calculation, and special orientation. Next, some prototypes designed for representing patient tasks are shown.

The eMental system provides users a greater possibility of easily access to the technology world and, at the same time, strengthens their mental functions with exercises that are displayed in a web environment. In addition, the system has an asynchronous mode, meaning that the patient performs exercises with the assistance of a family member or caregiver, anytime, anywhere. Furthermore, the system records the patient's activities to be evaluated by a doctor or therapist and the progress of the therapy is recorded visually.

As a result, the first design proposals obtained have an intuitive graphical interface that facilitates the interaction between the user and the system without having advanced computer skills. Moreover, each of the designed activities increases the self-esteem and reinforces the skills the patient still preserves, reducing frustration towards the therapy through encouraging messages to the successes and failures of the patient. This leads to improved results, such as increasing the attention of users and minimizing external distraction.

*(3) Implementation.* The implementation of the first version of the eMental system involved a hospital in the south of Spain. The hospital provided a rehabilitation team which consisted in *a medic, a physiotherapist, a patient, and a caregiver* and also furnished with areas for therapy rooms.

The multimedia engineering laboratory team was responsible for installing the technological artifacts (touchscreen, camera, and microphone) within the therapy rooms. Besides, they presented the proposal for the eMental system design by considering a detailed visualization of the tasks each patient should perform and the visualization process of the therapy each therapist should receive.

In order to evaluate the first design proposals, there were selected users who had the next features: (1) were familiar with cognitive impairment (such as doctors, therapists, patients, caregivers, and other specialists); (2) had basic computer skills, such as sending and receiving e-mail and capturing images from a camera or electronic device; (3) had knowledge of the test application, time availability, and exchange experiences on the issue; and finally (4) were interested in the rehabilitation through ICTs. The characteristics of the study group will be described in the following section.

The next step to the system is conducting tests with bigger groups, considering variables such as acceptance and usability.

*(4) Evaluation.* The researchers selected a group of potential users to analyze and evaluate the functional model designed from the prototypes improvements. A total of nine users attended the first presentation of functional layout of the system (two patients, two caregivers, one therapist, one doctor, two engineers, and one web designer).

To evaluate the usability of the system, first, the graphical interface and digitized exercises were presented. Later, the use and functionality of the system were explained in detail. Finally, verbal questions were performed to obtain each present person's point of view. As a result, on the one hand, small changes in the graphical interface (color, font size) were indicated. On the other hand, difficulty levels to the exercises were added.

At present, in order to improve the opportunity areas that have been identified in the first evaluation feedback, the system is in a redesign phase.

#### 4.1.2. Case Study 2: The e-Park System

The main objective of the e-Park system is the detection of cognitive deterioration of a person with Parkinson's disease. This is achieved through a telemedicine system that allows evaluating patients with a disease scale of PD-CRS by using telemedicine system.

*(1) Analysis.* Parkinson's disease has degenerative neurological symptoms that initially affect the motor system; however, it is evidenced that other areas could be affected by the progression of the disease, such as the cognitive and autonomic systems. The cognitive affectation is concentrated in the executive functions area, visuospatial skills and some forms of memory and language [40, 41]. The main symptoms of this disease are poor control of movements: shaking, sluggishness, stiffness, and abnormalities of posture and walking. Moreover, the affected person requires different cares to help him/her perform daily activities. The assistance of family members or caregivers for improving his/her life quality is required.

As a result of a first approach with a hospital in Spain, it was possible to define the participation of the different actors that were involved in a rehabilitation process.

The e-Park system is focused on the next users: doctors, patients, caregivers, and other specialists (as described in Section 4.1.1, Table 2). Next, the approaches obtained from the study are presented as follows:

- professional cases (doctors, therapists, and others specialists) require a telemedicine system that helps them monitor and trace patients with cognitive deterioration. It also should be able to manage a customized therapy sessions;
- patient cases require an asynchronously system that allows access to patient from any point and place without going to the hospital or rehabilitation place;
- its require system to allow remote patient monitoring and the evolution and implementation of medication is administered at home;
- the patients are those who have Parkinson's disease. This procedure includes supporting family and/or caregivers that assist in the rehabilitation of patients.

Same as the previous case study, to understand the needs and limitations of each user, a direct observation was used to understand their situation, environment, and activity. Then, all comments were taken into account in the system design process.

**Figure 5:**
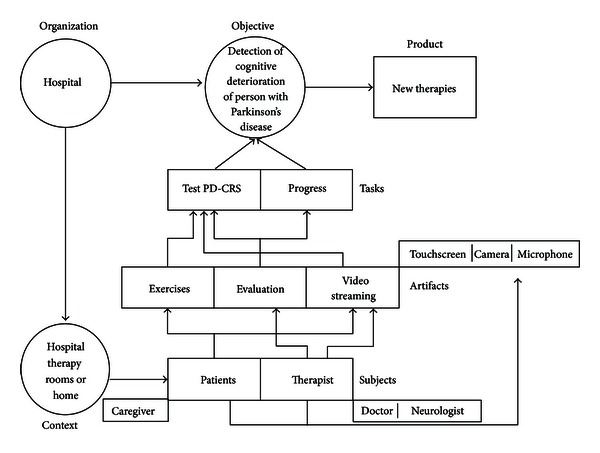
Representation of the therapy process for the e-Park system by following MAIA methodology.

After understanding the goals and needs of users, the next step consisted in organizing the information according to the MAIA framework [37]. In order to facilitate the understanding of the main actors involved in the therapeutic process and to allow further system development adapted to the actual needs and circumstances, the representation of the therapy process for the e-Park system by following MAIA methodology is shown (see Figure 5).

*(2) Design.* The e-Park system is planned by expert doctors experienced in specialized drugs treatment (the selection of drug treatment/therapy should be made after the patient with Parkinson's disease has been properly informed of drug efficacy [42]) for rehabilitation of Parkinson's disease. The e-Park system is designed for specialists in the application of the PD-CRS test. At the design and development stages, different roles were identified as follows: (a) the medical team defined the activities for the test; (b) the design team proposed the graphical user interface that attempted to represent communication between patient and specialist; and finally (c) the development team was responsible for programming and executing the contents of the system.

In order to characterize the contents of the system as far as possible, a conceptual design of the e-Park system was schematized after conducting a literature review. This process was conducted with the support of several medical specialists and taking into account the opinions and experiences gathered in the direct observation. In the traditional therapy, the therapist performs the therapy through using pencil and paper, supported with a test called *Cognitive Rating Scale (PD-CRS)*.

The e-Park system was designed in a way that the therapy can be performed by the patient in the comfort of his or her home. Each question on the test was adapted and customized within the system so that the therapist could easily record the results of their patients, achieving a reduction of costs and time for both the patient and the hospital. Besides, the patients could perform the exercises in a more comfortable and easily way.

The test (PD-CRS) includes nine divisions with a maximum total score of 92 as follows:

- subcortical functions: attention (10), short memory (10), working memory (10) and delayed memory (12), verbal fluency and alternating action, and spontaneous drawing of clock (10);
- cortical functions: designation (20) and a clock copy (10).

The verbal fluency and alternating do not have maximum scores. They are obtained by adding the rating for subcortical subscales, the cortical and the total PD-CRS. Better punctuation provides better cognitive level.

Once the components and contents were defined, the team performed several low-fidelity prototypes (sketches) through designing quick and easy user interface. These prototypes were presented to the medical team and the development team in order to get comments and new ideas regarding the design of the system framework.

**Figure 6:**
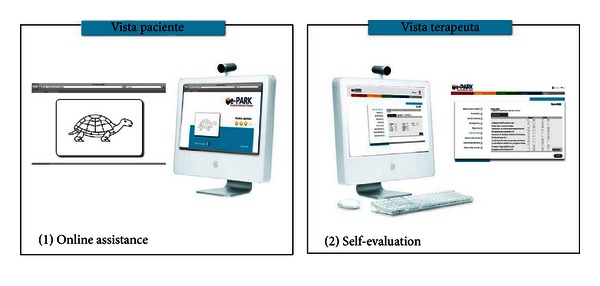
System application modes.

Therefore, a better designed high-fidelity prototype was obtained. Next, two application modes of the system are determined (see Figure 6):

1. online assistance, which means helping the specialist perform a real-time evaluation of the patient;
2. self-evaluation, meaning the evaluation of the patient himself or herself with this assistance of his or her family members and caregivers previously trained in this activity.

**Figure 7:**
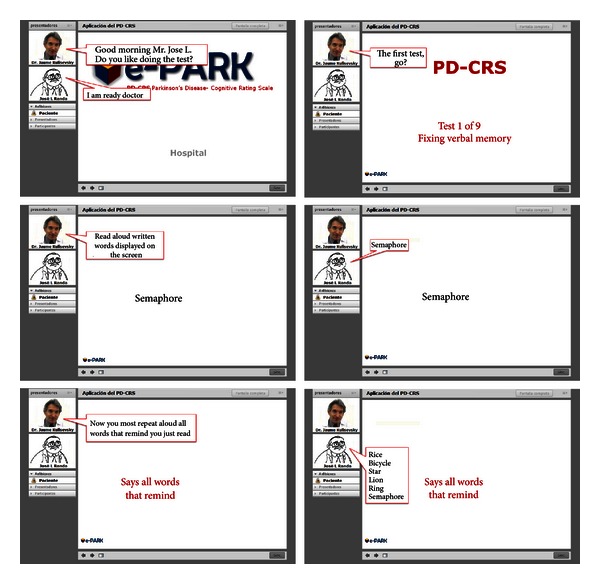
Sequence of the therapy.

The e-Park system has an asynchronous mode (remote real-time communication), meaning that the patient performs the test exercises with the assistance of a family member or caregiver, anytime and anywhere. Furthermore, it allows the specialist to manage the application of the system and their patients through using a web application such as video conferencing (see Figure 7).

The patient performs the test in front of a screen by following the specialist's instructions, and the specialist interacts with another application. This allows guiding the execution of the PD-CRS test and the record of the results of the patient.

*(3) Implementation.* The implementation of the first version of the e-Park System involved two hospitals in southern Spain. The hospitals provide a rehabilitation team, which consisted in a *medic, a therapist, a neurologist, a patient, and a caregiver *and also furnished with areas for therapy rooms. The multimedia engineering laboratory team was responsible for installing the technological artifacts (touch screen, camera, and microphone) within the therapy rooms. Besides, they presented the proposal of functional design by considering a detailed visualization of the test PD-CRS.

In order to evaluate the first design proposals, there were selected users who had the next features: (1) were familiar with Parkinson's disease (such as doctors, therapists, patients, caregivers, and other specialists); (2) had basic computer skills, such as sending and receiving email and capturing images from a camera or electronic device; (3) had knowledge of the test application, time availability, and exchange experiences of the issue; and finally (4) were interested in the rehabilitation through ICTs. The characteristics of the study group will be described in the following section.

The next step to the system is conducting tests with bigger groups, considering variables such as acceptance and usability.

*(4) Evaluation. *It is important to mention that eMental and e-Park systems are in a phase of evaluation and redesign. Because of this, this research shows the first results obtained in the usability evaluation of the two systems.

Qualitative evaluation is used in the research. Therefore, the number of participants in the case study is small. Furthermore, the qualitative analysis allows the data to be manipulated in order to extract a relevant significance regarding the objective of the research.

The patients were selected for a consecutive period of 12 weeks between April 2010 and July 2010. Appropriate cases were defined to those following patients:

- patient 1: patient with primary Parkinson's disease;
- patient 2: patient with primary Parkinson's disease;
- patient 3: patient in the predementia stage of Alzheimer's disease;
- patient 4: patient in the early stage of Alzheimer's disease.

The age range for this study is 60 years old, with only one case being under the age of 50 years. The rate for men is 75% and for women 25%.

The work team showed the functional prototype of the eMental system. The doctors and therapists visualized the tool by running the system and exercises of the patients. 

Moreover, the work team showed the functional prototype of the e-Park system. The patient performed the test in front of a screen by following the specialist's instructions, and the specialist interacted through another application.

Finally, the complete evaluation of the first proposal of the two systems took place on February 24, 2011. It involved a total of 17 people, including four patients, four caregivers, two therapists, one neurologist, three doctor, two engineers, and one web designer.

In order to carry out a first evaluation of the two systems, a questionnaire based on parameters of usability and technology acceptance (the technology acceptance evaluates a series of factors that influence the decision on *how* and *when* the patient will use the technology [43]) was applied. The questionnaire composed of 16 closed questions was answered by the studying group. The mean time of the evaluations duration was approximately 30 minutes.

The questionnaire applied to the study group in order to get information about parameters of usability and acceptance of technology is shown in Table 3.

As shown previously, the questionnaire is composed of 15 closed questions, grouped into five parameters: easiness of navigation, learnability, satisfaction, operability, and functionality. The questionnaire also included one open question in order to obtain different comments and feedback of the system.

The scale of measure used in the questionnaire corresponds to a Likert scale of seven points. The options in the scale was as follows: (a) the range of values between 1 and 3 were selected for expressing disagreement; (b) the value 1 was selected for strong disagreement; (c) the value 4 was selected for a neutral opinion; and finally (d) the range of values between 5 and 7 was selected for expressing agreement, where 7 meant totally agree.

**Figure 8:**
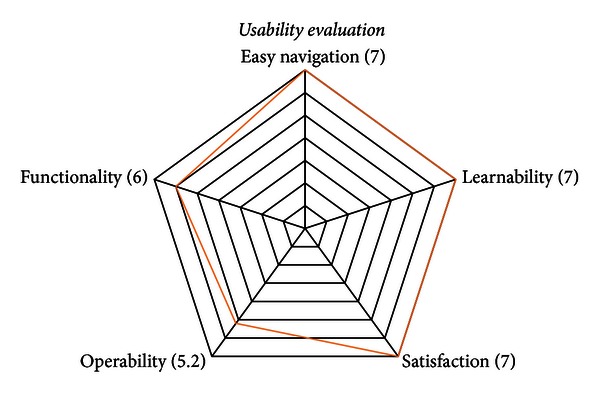
The results obtained of usability evaluation.

Then, a total of 16 validated questionnaires were obtained. The rate of response of the questionnaires was 82.35% (referred to the total percentage of medical personnel and patients in the hospital). The summary of results obtained from the application of the questionnaire is shown below (see Figure 8). 

In the analysis of the first parameter, *ease of navigation*, the users showed a positive agreement (7), asserting that telemedicine systems designed are of easy navigation, and therefore, the patients will be able to use it at home. A positive value in the first parameter finally promotes the implementation of telemedicine systems for the rehabilitation of patients with neurological diseases.

In the second parameter, *learnability*, the users showed a positive agreement (7), reflecting to agree on the design of telemedicine systems is friendly, clear, and easy to understand. This indicates the systems designed in this telemedicine research taking into consideration the needs of the hospital and patients.

The third analyzed parameter is *satisfaction*; the user showed a favorable result (7), manifesting a positive agreement that telemedicine systems provide an attractive environment for rehabilitation.

In the parameter of *operability*, there is a very minimal margin between the answers provided (5.2), which indicates that in the future, the users need more time to evaluate the usability of the system.

Concerning the analysis of the last parameter, functionality, the users showed a positive agreement (6), affirming that the design of therapy allowed the patients to perform rehabilitation exercises in a more easy and attractive way.

### 4.2. Technological Attributes

Table 4 shows a comparative analysis according to the technological attributes that are provided by the telemedicine systems developed in this research.

This comparison shows that the systems described in this research are designed step by step, with the purpose of customizing therapies according to the profile and disability of patients, taking into account the needs of patients throughout the system design process.

## 5. Conclusions

Telemedicine is gradually, if not rapidly, becoming a technological and clinical reality. Therefore, it is essential to address the challenge that exists in the successful evaluation of a telemedicine system. By reviewing the literature related to telemedicine systems, we noticed the necessity to concentrate on the specific user requirements, particularly referring to patients, in order to develop an intuitive and effective system.

This research work proposes a framework based on the UCD methodology and MAIA framework, to design systems customized to particular users with specific characteristics and needs, increasing the acceptance and satisfaction in the users. An important feature of the proposed framework is the involvement of end users through the design processes, which allows collecting important data of user needs. Moreover, the reliability and validation of the first functional layouts are increased. Therefore, this framework becomes the source to optimizing the design of the telemedicine systems interfaces according to users' real needs. The proposed framework is composed of four phases: analysis, design, implementation, and evaluation.

Besides, this paper shows the performance of the proposed framework through implementing it in two case studies to design two systems: eMental system and e-Park system. These telemedicine systems were developed in real hospital environments in order to focus on monitoring the rehabilitation of patients with neurological disorders. All designed telemedicine systems were customized to cognitive and physical limitations of the patients. This way, they provided visual information of patient progress to the medical staff.

As main benefits, this approach reduces significantly the time of patients in hospitals, without reducing the continuous monitoring of patients. Moreover, it facilitates the flexible interaction between patient and doctor through using web application. This shows the efficiency of using the proposed framework to design telemedicine systems. 

After implementing the proposed framework to design the two systems, we can conclude that, to achieve improvements in quality health care and to reduce errors, researchers and system developers must work together to integrate the knowledge of user-centered design toward the design of new systems customized to users with specific needs. Moreover, there is a need of collaboration among medical team, the design team, and the development team to ensure a well design and perform of telemedicine systems.

### 5.1. Future Work

This work proposes the adoption of user-centered design (UCD) methodology for designing and developing telemedicine systems in order to support the rehabilitation of patients with mental deficiencies. Then, four research lines are identified from this research:

1. deploy the proposed framework in other telemedicine systems and include other related technology, in order to identify more findings and get more favorable results;
2. develop tailored versions of telemedicine system for mobile devices;
3. implement the proposed approach in the treatment and rehabilitation therapy file;
4. incorporate intelligent agents to support the patient and medical staff in telemedicine systems.

## Figures and Tables

**Table 1:** Advantages and disadvantages with respect to other systems developed.

Systems	Description of system	Advantage	Disadvantages
The RuralHub Telepsych system [44]	Report on a geriatric telepsychiatry consultation service provided by a tertiary-care hospital to rural nursing homes located up to a few hours' drive away. It has been successfully used with a wide variety of diagnostic groups (such as patients with depression, posttraumatic stress disorder, panic disorder and/or agoraphobia, Alzheimer's disease, schizophrenia, and other mental-health conditions).	(i) Patients show acceptance and adherence to a treatment regimen. (ii) Telepsych is comparable to conventional treatment in outcomes and cost. (iii) Overcomes the traditional therapy (face-to-face sessions), particularly when dealing with patients prone to violence or who are afraid of leaving home for treatment.	(i) It creates “an impersonal atmosphere.” (ii) Is problematic for elderly patients with sensory impairments, for treating uncooperative or paranoid patients, and in emergency situation. (iii) Telepsych does not have a collaborative online environment to support exchange of formal and informal information. (iv) There is not involvement of users in the design and development stages of the system.

Portable tele-assessment system [45]	Remote evaluation of the elbow joint with spasticity and contracture in patients with neurological disorders. Especially in patients with spasticity.	(i) Provided physical as well as audiovisual interaction between the clinician and the patient. (ii) Saving time and costs involved in the rehabilitation. (iii) Attractive to the patients since it was designed to be lowcost and portable. (iv) Allows remote monitoring of a remote way the progression of physical treatment without missing the essential part—physical feel.	(i) The spasticity test is limited, because the doctor has to perform therapy exercises, burn for teaching, and the replay to be viewed by the patient. (ii) There is not involvement of users in the design and development stages of the system.

AUBADE system [46]	AUBADE is an integrated platform built for the affective assessment of individuals. The system performs the evaluation of the emotional state.	(i) It has an intelligent emotion recognition module and a facial animation module. (ii) It has databases where the acquired signals along with the subject's animation videos are saved. (iii) Is a multifunctional system that can be utilized in many different ways and in multiple application fields. (iv) The AUBADE system consists of a multisensorial wearable, a data acquisition, and wireless communication module, a feature extraction module.	(i) The system's clinical application is based on the ability of supporting clinical diagnosis related to all the pathologies taking into account if the patient's capability to feel and express emotions is limited or totally absent. (ii) Due to the fact that emotions vary from person to person, the system must be trained by using a variety of subjects and then by testing its performance; this implies an investment of time that often people and specialists do not have.

Telemedical Interventional Monitoring in Heart Failure (TIM-HF) [47, 48]	Wireless Bluetooth system with a personal digital assistant (PDA) that performs automated encrypted transmission via mobile phone of electrocardiogram measurement, blood pressure measurement, and body weight. The telemonitoring system consists, on the one hand, of portable home devices (ECG, and blood pressure measurement and body weight) connected to PDA via a local network. On the other hand, a telemedical workstation with electronic patient record that is a web application with a graphical user interface browser so that incoming measurements generate events according to a set of medical prioritization rules to initiate a workflow-guided review process in the telemedical workstation and its further evaluation by medical professionals.	(i) Prevents hospitalizations by early detection of disease worsening followed by immediate intervention. (ii) Home devices for ECG, body weight, blood pressure, and self-assessment measurement are used. (iii) PDA has a touchscreen option for a scaled self-assessment. (iv) A home emergency call system provides direct contact to health-care professionals. (v) The medical system has been built as an open platform to integrate other home devices for monitoring such as diabetes, chronic obstructive pulmonary disease, anticoagulants, and implantable cardiac device information.	(i) Telemedical centers must operate around the clock every day of year because it requires immediate diagnosis and prompt treatment. (ii) To ensure patient safety, it was required at least that 94% of the total system be available, including the mobile phone network. (iii) Wireless technology is susceptible to faults. Besides there is not defined standards for secure wireless transmission of the data. (iv) The volunteers that proved the system were younger than the anticipated chronic heart population.

Heart failure case disease management program [49]	Is a care process that verifies the state of a patient illness throughout sending the information concerning his or her vital signs such as weight, systolic blood pressure, heart rate, dyspnoea, asthenia, edema, therapy changes, blood urea nitrogen, creatinine, sodium, potassium, and bilirubine to medical staff in order to support decision-making to prevent haemodynamic imbalance, to reinforce educational support, to optimize therapy, and to improve quality of life and outcomes. The telemedical staff and patients use a touchpad or mobile phone at their home, after having dialed a toll-free number. Then, each parameter was entered in reply to question asked by a recorded voice and a confirmation was requested to each of them.	(i) The program uses a toll-free number. (ii) The overall procedure is managed by an interactive voice response (IVR) system (Appel Electtronica Srl, Turin); therefore, the data transmission did not require operator support. (iii) The daily telemonitoring activities typically began by listening vocal message and taking the appropriate actions. (iv) Optimized therapy and continuous redefinition of the care process. (v) Increases patient's knowledge about management of illness, recognition of initial signs, and symptoms. (vi) The tight relationship between health-care personnel and patients allows coaching in a way that the patient is simulated toward an activate participation in self-management of his or her illness.	Management effectiveness depends on the team management, the intensity of treatment, the parameters monitored, the standardization of managerial algorithms and the characteristics of the patients.

eMental System*	Supports the rehabilitation of the elderly with cognitive impairment through promoting social integration. It provides a cognitive stimulation therapy to the patients, caregivers, and specialists.	(i) It manages automatically the degree of difficulty to suit the cognitive level of each patient. (ii) Provides visual feedback to users' tasks. (iii) The therapy is performed in the comfort (iv) of home. (v) It promotes family to incorporate with patient rehabilitation. (vi) Saving time and costs involved in rehabilitation.	

e-Park system*	The detection of cognitive deterioration of person with Parkinson's disease. By applying the PD-CRS test through Internet.	(i) Standardization and optimization in the application of the PD-CRS test. (ii) Capture and execution of quasi-digital PD-CRS test. (iii) The system automatically manages the patients and the time duration for each session. (iv) Provides visual feedback of the test results.	The first version of the system is not depending on the limitations of the patients.

*Telemedicine system described in this research.

**Table 2:** Descriptions of the user types.

User types	Function
Patient	(i) Requests help the caregiver. (ii) Performs activities assigned by the therapist.

Caregiver	(i) Provides patient instruction. (ii) Encourages the patient. (iii) Gives feedback to the patient.

Therapist	(i) Assigns therapy activities. (ii) Provides indications to the caregiver. (iii) Performs assessment of patients with the results obtained in each of the assigned activities. (iv) Facilitates feedback and helps the patient.

Doctor	(i) Provides indications to the therapist. (ii) Provides medical evaluation of patient.

Administrator	Manages system resources

**Table 3:** Questions applied to the study group. Adaptation of Martínez Alcalá, 2012 [50].

Parameter	Questions
Easiness of navigation	(i) Is my interaction with telemedicine system clear and understandable? (ii) Was it easy for me to use the telemedicine system? (iii) Do I find easy to use the telemedicine system?

Learnability	(i) Is it easy for me to learn how to operate the telemedicine system? (ii) Is the telemedicine system design friendly? (iii) Are the instructions given in therapy clear and easy to understand?

Satisfaction	(i) Is it helpful to implement a telemedicine system for rehabilitation? (ii) Is the telemedicine system design an attractive idea? (iii) Does the telemedicine system provide an attractive rehabilitation environment? (iv) Do I like working with telemedicine system?

Operability	(i) Does the telemedicine system design meet your expectations? (ii) Does the design (appearance, color, shape, etc.) clearly show that it is a support system to improve the cognitive impairment problem? (iii) Is the telemedicine system capable of correctly representing improvements experienced by the treatment? (iv) Do I find the telemedicine system useful in my rehabilitation?

Functionality	Does the design of therapy allow its activities easier?

Open question	In general, do you think that undergoing a rehabilitation process through the telemedicine system increases the chances of improving your current condition?

**Table 4:** Comparison of systems technological attributes.

	Devices	Compatibility	Complexity	Accessibility	Portability	Satisfaction and acceptance
(1) The RuralHub Telepsych system	E-mail, fax, and telephone	X	X	—	—	X
(2) Portable teleassessment system	Cameras, microphones, and PC	X	X	—	—	—
(3) AUBADE system	Integrated platform and PC	X	—	—	X	X
(4) Telemedical interventional monitoring in heart failure	Personal digital assistant, home devices (ECG), and Web application	—	X	—	X	X
(5) Heart failure case disease management program	Touchpad or mobile phone	X	—	X	X	X
(6) eMental system	Web application, cameras, microphone, and touchscreen	X	X	X	X	X
(7) e-Park System	Web application, cameras, microphone, and touchscreen.	X	X	X	X	X

## References

[1] Shiferaw F, Zolfo M (2012). The role of information communication technology (ICT) towards universal health coverage: the first steps of a telemedicine project in Ethiopia. *Glob Health Action*.

[2] Li J, Westbrook J, Callen J, Georgiou A (2012). The role of ICT in supporting disruptive innovation: a multi-site qualitative study of nurse practitioners in emergency departments. *BMC Medical Informatics & Decision Making*.

[3] Teixeira L, Ferreira C, Santos BS (2012). User-centered requirements engineering in health information systems: a study in the hemophilia field. *Computer Methods and Programs in Biomedicine*.

[4] Borowski M, Siebig S, Wrede C, Imhoff M (2011). Reducing false alarms of intensive care online-monitoring systems: an evaluation of two signal extraction algorithms. *Computational and Mathematical Methods in Medicine*.

[5] Rusu M, Saplacan G, Sebestyen G, Todor N, Krucz L, Lelutiu C (2010). eHealth: towards a healthcare service-oriented boundary- less infrastructure. *International Journal of Telemedicine and Applications*.

[6] Galán-Retamal C, Garrido-Fernández R, Fernández-Espínola S, Padilla-Marín V (2010). Monitoring polymedicated elderly patients in a health care unit. *Farmacia Hospitalaria*.

[7] Wootton R, Geissbuhler A, Jethwani K (2012). Long-running telemedicine networks delivering humanitarian services: experience, performance and scientific output. *Bulletin of the World Health Organization*.

[8] Wade VA, Karnon J, Elshaug AG, Hiller JE (2010). A systematic review of economic analyses of telehealth services using real time video communication. *BMC Health Services Research*.

[9] Kurki M, Hätönen H, Koivunen M, Anttila M, Välimäki M (2012). Integration of computer and Internet-based programmes into psychiatric out-patient care of adolescents with depression. *Informatics for Health and Social Care*.

[10] Melas CD, Zampetakis LA, Dimopoulou A, Moustakis V (2011). Modeling the acceptance of clinical information systems among hospital medical staff: an extended TAM model. *Journal of Biomedical Informatics*.

[11] Lin C, Lin IC, Roan J (2012). Barriers to physicians’ adoption of healthcare information technology: an empirical Study on multiple hospitals. *Journal of Medical Systems*.

[12] Bernard R, McNeil S, Cook D, Agarwal K, Singhal GR (2011). Preparing for the changing role of instructional technologies in medical education. *Academic Medicine*.

[13] Ruxwana NL, Herselman ME, Conradie DP (2010). ICT applications as e-health solutions in rural healthcare in the Eastern Cape Province of South Africa. *Health Information Management Journal*.

[14] Ruxwana N (2009). *Technology Assessment of Rural Hospitals in the Eastern Cape Province: Knowledge, Adoption, Access, and Availability of E-Health Solutions for Improved Health Care Services Delivery in Rural Hospitals*.

[15] Borges NJ, Navarro AM, Grover A, Hoban JD (2010). How, when, and why do physicians choose careers in academic medicine? A literature review. *Academic Medicine*.

[16] Veneri D (2011). The role and effectiveness of computer-assisted learning in physical therapy education: a systematic review. *Physiotherapy Theory and Practice*.

[17] Barneveld Binkhuysen FH, Ranschaer ER (2011). Teleradiology: evolution and concepts. *European Journal of Radiology*.

[18] Sorknæs AD, Madsen H, Hallas J, Jest P, Hansen-Nord M (2011). Nurse tele-consultations with discharged COPD patients reduce early readmissions—an interventional study. *Clinical Respiratory Journal*.

[19] Pawar P, Jones V, van Beijnum BJF, Hermens H (2012). A framework for the comparison of mobile patient monitoring systems. *Journal of Biomedical Informatics*.

[20] Peirce SC, Hardisty AR, Preece AD, Elwyn G (2011). Designing and implementing tele- monitoring for early detection of deterioration in chronic disease: defining the requirements. *Health Informatics Journal*.

[21] Reis CI, Freire CS, Fernández J, Monguet JM (2011). Patient centered design: challenges and lessons learned from working with
health professionals and schizophrenic patients in e-therapy contexts. *Communications in Computer and Information Science*.

[22] Ayed MB, Ltifi H, Kolski C, Alimi AM (2010). A user-centered approach for the design and implementation of KDD-based DSS: a case study in the healthcare domain. *Decision Support Systems*.

[23] Palmas W, Shea S, Starren J (2010). Medicare payments, healthcare service use, and telemedicine implementation costs in a randomized trial comparing telemedicine case management with usual care in medically underserved participants with diabetes mellitus (IDEATel). *Journal of the American Medical Informatics Association*.

[24] Aanesen M (2011). Smarter elder care? A cost-effectiveness analysis of implementing technology in elder care. *Health Informatics Journal*.

[25] Butler TN, Yellowlees P (2012). Cost analysis of store-and-forward telepsychiatry as a consultation
model for primary care. *Telemedicine Journal and E-Health*.

[26] Wallach D, Scholz SC (2012). User-centered design: why and how to put users first in software development. *Software for People Management for Professionals*.

[27] Gardiner S, Hartzell T (2012). Telemedicine and plastic surgery: a review of its applications, limitations and legal pitfalls. *Journal of Plastic, Reconstructive & Aesthetic Surgery*.

[28] Raju PK, Prasad S (2012). Telemedicine and cardiology decade of our experience. *Journal of Indian College of Cardiology*.

[29] Bynum AB, Irwin CA (2011). Evaluation of the effect of consultant characteristics on telemedicine diagnosis and treatment. *International Journal of Telemedicine and Applications*.

[30] Fong B, Fong ACM, Li CK (2010). *Telemedicine Technologies: Information Technologies in Medicine and Telehealth*.

[31] While A, Dewsbury G (2011). Nursing and information and communication technology (ICT): a discussion of trends and future directions. *International Journal of Nursing Studies*.

[32] L E (2010). Telemedicine: the future of outpatient therapy?. *Clinical Infectious Diseases*.

[33] Rogove HJ, McArthur D, Demaerschalk BM, Vespa PM (2012). Barriers to telemedicine: survey of current users in acute care units. *Telemedicine and E-Health*.

[34] Kowitlawakul Y (2011). The technology acceptance model: predicting nurses’ intention to use telemedicine technology (eICU). *Computers Informatics Nursing*.

[35] Ekeland AG, Bowes A, Flottorp S (2010). Effectiveness of telemedicine: a systematic review of reviews. *International Journal of Medical Informatics*.

[36] Ghorbani A, Dignum V, Dijkema G (2012). An analysis and design framework for agent-based social simulation. *Advanced Agent Technology*.

[37] Ferruzca Navarro MV (2008). *Estudio teórico y evidencia empírica de la aplicación del marco teórico de “Cognición Distribuida” en la gestión de sistemas de formación e- Learning [Tesis Doctoral]*.

[38] Cole E Patient-centered design: interface personalization for individuals with brain injury.

[39] Pavliha D, Reberšek M, Miklavčič D (2011). A graphical user-interface controller for the biomedical
high-voltage signal generator. *Journal of Electrical Engineering and Computer Science*.

[40] Shulman JM, Jager P, Feany MB (2011). Parkinson’s disease: genetics and pathogenesis. *Annual Review of Pathology*.

[41] Weintraub D, Koester J, Potenza MN (2010). Impulse control disorders in Parkinson disease: a cross-sectional study of 3090 patients. *Archives of Neurology*.

[42] Unlu E, Cevikol A, Bal B, Gonen E, Celik O, Kose G (2010). Multilevel botulinum toxin type a as a treatment for spasticity in children with cerebral palsy: a retrospective study. *Clinical Science*.

[43] Šumak B, Heričko M, Pušnik M, Polančič G (2011). Factors affecting acceptance and use of moodle: an empirical study based on TAM. *Electrical Engineering*.

[44] Hansen SW, Gogan JL, Baxter RJ Distributed cognition in geriatric telepsychiatry.

[45] Park HS, Wu YN, Ren Y, Zhang LQ A tele-assessment system for evaluating elbow spasticity in patients with neurological impairments.

[46] Katsis CD, Ganiatsas G, Fotiadis DI (2006). An integrated telemedicine platform for the assessment of affective physiological states. *Diagnostic Pathology*.

[47] Koehler F, Winkler S, Schieber M (2010). Telemedical Interventional Monitoring in Heart Failure (TIM-HF), a randomized, controlled intervention trial investigating the impact of telemedicine on mortality in ambulatory patients with heart failure: study design. *European Journal of Heart Failure*.

[48] Winkler S, Schieber M, Lücke S (2011). A new telemonitoring system intended for chronic heart failure patients using mobile telephone technology—feasibility study. *International Journal of Cardiology*.

[49] Capomolla S, Pinna G, La Rovere MT (2004). Heart failure case disease management program: a pilot study of home telemonitoring versus usual care. *European Heart Journal, Supplement*.

[50] Martínez Alcalá C (2012). *Modelo de vistas personalizadas para la gestión de contenido en comunidades I+D+i. [Tesis Doctoral]*.

